# Transcapillary fluid flux and inflammatory response during neonatal therapeutic hypothermia: an open, longitudinal, observational study

**DOI:** 10.1186/s12887-018-1020-3

**Published:** 2018-02-23

**Authors:** Hans Jørgen Timm Guthe, Torbjørn Nedrebø, Jan Kristian Damås, Helge Wiig, Ansgar Berg

**Affiliations:** 1 Department of Pediatrics and Adolescent Medicine, Haukeland University Hospital, Bergen, Norway; 2 Department of Clinical Science, University of Bergen, Bergen, Norway; 3 Surgical Department, Haraldsplass Deaconess Hospital, Bergen, Norway; 4 Centre of Molecular Inflammation Research, Department of Cancer Research and Molecular Medicine, Norwegian University of Science and Technology, Trondheim, Norway; 5 Department of Infectious Diseases, St. Olav’s Hospital, Trondheim, Norway; 6 Department of Biomedicine, University of Bergen, Bergen, Norway

**Keywords:** Neonate, Osmotic pressure, Edema, Asphyxia, Therapeutic hypothermia, Inflammation, Cytokines

## Abstract

**Background:**

Therapeutic hypothermia is neuroprotective in asphyxiated neonates by counteracting mechanisms contributing to brain injury. Although an initial increased permeability is part of an inflammatory reaction and thereby a natural healing process, an excessive endothelial permeability with edema formation may result in impaired hemodynamics. Reduced permeability may, however, benefit healing. Although plasma and interstitial colloid osmotic pressure are accessible and essential parameters for understanding fluid imbalance, the mechanisms of fluid exchange remain poorly understood. The potential influence of therapeutic hypothermia on plasma and interstitial colloid osmotic pressure, and the relationship between inflammatory markers and colloid osmotic pressure in asphyxiated neonates, was investigated.

**Methods:**

Seventeen neonates with moderate to severe hypoxic ischemic encephalopathy, born after 35 weeks gestation, received servo-controlled whole body cooling before 6 h of age, followed by gradual rewarming after 72 h. All infants were treated according to a national hypothermia protocol. Interstitial fluid in the skin was collected at 7, 13, 25, 49, and 73 h after birth by subcutaneous implantation of multifilamentous nylon wicks with 60 min of implantation time. Biomarkers of inflammation and colloid osmotic pressure were measured in serum and interstitial fluid.

**Results:**

A modest decrease in serum and interstitial colloid osmotic pressure was measured, leaving an unaltered difference in colloid osmotic pressure gradient. A decline in mean arterial pressure was observed between 7 and 13 h of life, with a concomitant decrease in positive fluid balance within the same time frame. White blood cell count and leukocyte subclasses dropped significantly throughout treatment, with elevated interstitial interleukin (IL)-1α and decreased serum IL-1RA, IL-6, and IL-10 during treatment time points.

**Conclusions:**

Colloid osmotic pressures measured in serum and interstitial fluid during asphyxia is lower than previously reported, with small alteration of pressure differences across capillaries, reducing vascular filtration. An inherent local and systemic regulation of inflammation together with changes in colloid osmotic pressure may indicate a possible preventive mechanism of edema generation during neonatal asphyxia and therapeutic hypothermia.

**Trial registration:**

ClinicalTrials.gov Identifier: NCT01044940. Date of registration: January 8, 2010.

## Background

Hypoxic ischemic encephalopathy (HIE) due to perinatal asphyxia is an important cause of childhood neurodevelopmental deficits that include loss of motor and cognitive functions [1, 2]. Hypoxic ischemia could be involved in the injuries correlating with HIE by initiating a detrimental inflammatory response, partly through the release of reactive oxygen species and amplified by inflammatory cytokines. Several clinical studies and reports from experimental models have shown that immune pathogenic mechanisms may play an essential role in HIE by affecting both the central nervous system and systemic circulation [3–6]. Therapeutic hypothermia (TH) induces an artificial reduction of core and brain temperature to 33.5 **°**C. This results in reduced cerebral and whole-body metabolism, followed by attenuation of oxidative stress after hypoxemia [7]. Several studies have shown that TH also provides neuroprotection in neonates with moderate to severe HIE [8, 9], potentially through favorable effects on multiple pathways contributing to brain injury. Thus, TH has been demonstrated to induce an anti-inflammatory response with reduced amounts of circulating leukocytes and chemokines in neonates with HIE, as well as in animal models [5, 10]. This inhibiting effect of the immune system in response to TH may be protective against the over-functioning immune reaction observed in these children [11]. Moreover, hypothermia has been shown to have beneficial effects on capillary integrity, as evidenced by reduced accumulation of interstitial fluid (IF) through decreased endothelial permeability, in rats after hemorrhagic shock [12]. However, increased intravascular hydrostatic pressure, due to TH-induced peripheral vasoconstriction, along with reduced venous return [13], could theoretically result in interstitial accumulation of fluid. Few studies have addressed these mechanisms of fluid imbalance during TH in neonates, and cytokine concentrations in the cellular microenvironment have not been previously measured. Because plasma colloid osmotic pressure (COP_p_) and interstitial colloid osmotic pressure (COP_i_) are essential determinants of fluid exchange during edema formation [14], we herein examined whether TH influenced these parameters in neonates with HIE, expecting a reduced COP_p_ as reported in sick neonates [15]. We also explored the association between COP_p_ and COP_i_ and inflammatory processes during TH by measuring a wide panel of cytokines in the plasma and IF.

## Methods

### Ethics

The study was approved by the Regional Committees for Medical and Health Research Ethics, Western Norway, and was conducted at the Neonatal Intensive Care Unit (NICU), Haukeland University Hospital, Bergen, Norway. All patients were included in the study following written informed consent obtained from a parent or guardian after explanation of the study in accordance with the Declaration of Helsinki. The study trial was registered in ClinicalTrials.gov (ClinicalTrials.gov Identifier: NCT01044940).

### Study population

The study was an open, longitudinal, observational study, taking place between June 2009 and October 2014. Newborns treated with TH owing to moderate and severe HIE were included after fulfillment of national inclusion criteria for neonatal TH. Inclusion and exclusion criteria are presented in Table 1.

**Table 1:** Criteria for inclusion of therapeutic hypothermia

Inclusion criteria	Exclusion Criteria
A: Gestational age ≥ 36 weeks	Expected need for surgical treatment (before 3 days of age)
B: At least one of the following:	Severe birth defects with expected poor prognosis
1. Apgar score ≤ 5 at 10 min after birth	
2. Requires positive pressure ventilation 10 min after birth	
3. pH < 7.00 in umbilical arterial blood or arterial blood within 60 min after birth	
4. Base excess ≤ − 16 in umbilical arterial blood or arterial blood within 60 min after birth	
C: Signs of moderate to severe encephalopathy with at least one of the following:	Age > 6 h before hypothermia could be initiated
1. Hypotonia	
2. Abnormal reflexes or constricted/deviated, dilated, nonreactive to light pupils	
3. Weak or absent sucking reflex	
4. Presence of seizures	

### Clinical management

All recruited patients were, as part of the clinical routine, sedated with morphine or fentanyl and intubated after delivery or before transport from referring hospitals at the attending physicians discretion. Rectal temperature was continuously monitored, with a target body temperature between 34 and 36 °C before TH. All patients received prophylactic antibiotics (penicillin and gentamycin) throughout TH, and scheduled clinical and biochemical monitoring was performed by the attending physician discretion.

### Neonatal TH protocol

TH was conducted according to the hospital hypothermia protocol. Induction and maintenance of hypothermia was achieved by servo-controlled whole body cooling equipment (Criticool; Mtre, Yavne, Israel) and the core temperature was reduced to 33.5 °C before 6 h of age and maintained until 72 h after birth. The rectal temperature was not allowed to increase more than 0.3 °C per hour during rewarming. After rewarming, rectal temperature was monitored for 24 h to prevent rebound hyperthermia. Patients with the expected poorest prognosis (in extremis) underwent magnetic resonance imaging (MRI) during cooling (second or third day of TH). Otherwise, imaging took place at day 4 to 7. Cerebral ultrasound was conducted during the first 3 days and before discharge.

### Intensive care monitoring and treatment

Patients were treated in an open-radiant infant warmer incubator (Dräger Babytherm 8010, Dräger Medical, Lübeck, Germany) with the heating blanket and radiant warmer turned off. TH data were recorded according to local protocols and monitoring was performed (Intellivue MP70 Neonatal, Philips, Eidhoven, Nederland) with continuous arterial pressure, oxygen saturation, heart rate, and respiration registration. All patients were monitored with an amplitude-integrated electroencephalogram to assess cerebral function within 3–6 h after TH initiation. From January 2011 onward, ventilation therapy was provided by Dräger Babylog® VN500 (Dräger Medical, Lübeck, Germany) with the pressure- and assist-controlled, volume-guaranty mode. Prior to 2011, the Stephanie Neonatal and Paediatric ventilator (Stephan, Gackenback, Germany) was utilized, with the pressure-controlled synchronized intermittent mandatory ventilation mode. Morphine sedation was administered throughout treatment if the patient showed signs of improvement during TH. Otherwise, if expected poor prognosis, sedation was halted and early MRI was performed. All neonates received the recommended reduced fluid intake [16] of approximately 40 ml/kg/day adjusted to weight, urine output, and serum sodium levels. Intravenously administered fluids consisted of isotonic saline (NaCl 9 mg/ml), glucose (concentration range of 100 mg/ml to 300 mg/ml, adjusted to a calculated need of 6 mg/kg/min), and blood products according to the patients’ requirements. Enteral feeding was not established during TH. The calculation of fluid balance was based on the total amount of fluid intravenously administered and measurements of hourly diuresis (urinary catheter) and gastric aspiration. Daily weight was not measured due to attached technical equipment, and fluid loss from respiratory evaporation or obvious fluid accumulation during TH was deemed not feasible due to the clinical setting. If the mean arterial pressure (MAP) was < 40 mmHg, and a fluid load of 9 mg/ml NaCl (10–20 ml/kg) proved ineffective, dopamine was administered. In the case of no clinical improvement, treatment was followed by adrenaline. Seizures during TH were treated with phenobarbital/phenytoin, and hypothermia was prolonged if the seizures appeared during rewarming.

### Sampling of interstitial fluid and blood

Sterile, nylon, multifilament wicks soaked in 9 mg/ml NaCl were subcutaneously implanted 4 cm in length into either the arm or the leg for 60 min in accordance with previous studies [14, 17]. The wicks were implanted at 6, 12, 24, 48, and 72 h after birth. Retracted wicks were placed into 1.5-ml Eppendorf centrifuge tubes with funnel (Sarstedt, Nümbrecht, Germany) containing mineral oil. Following centrifugation of the wicks, IF was extracted and frozen at − 20 °C for later analysis. Colloid osmotic pressure (COP) was measured using a colloid osmometer designed for small samples [18] equipped with a membrane impermeable for molecules > 30 kDa (PM-30, Amicron, Lexington, MA, USA). COP was amplified and recorded with Easy Graph P930 (Gould Inc., Ohio, USA). Blood samples were collected from an arterial line both before installation and retraction of the wick. The blood samples were allowed to clot and serum was subsequently isolated, frozen at − 20 °C. COP determined from either plasma or serum is equivalent [19], and patient serum, a surrogate for patient circulating plasma, was later analyzed in the above-mentioned colloid osmometer and is denoted in the manuscript as COP_p_. Additional blood analyses, such as serum albumin and hemoglobin concentrations, were performed at the Laboratory of Clinical Biochemistry at Haukeland University Hospital. The concentrations of 15 cytokines were analyzed in serum and IF using a multiplex bead immunoassay (Milliplex HCYTMAG-60; Merck Millipore, Darmstadt, Germany): interferon gamma, interleukin (IL)-1α, IL-1β, interleukin-1 receptor antagonist (IL-1RA), IL-2, IL-6, IL-8, IL-10, IL-12p40, monocyte chemoattractant protein-1, macrophage inflammatory protein-1α, transforming growth factor-α, tumor necrosis factor (TNF)-α, TNF-β, and vascular endothelial growth factor. The procedure was performed as recommended by the manufacturer, measured in a 1:12.5 dilution, and analyzed, with a Luminex 100™ instrument (Luminex Corp.; Austin, USA) and StarStation software (Applied Cytometry Systems; Dinnington, UK) at the Broegelmann Research Laboratory, Department of Clinical Sciences, University of Bergen, Norway. Values were reported as picograms per milliliter (pg/ml). Because of scarce amounts of material, we were not able to verify cytokine results by running the tests in parallel.

### Statistical analysis

Data and analysis were evaluated using SigmaStat 11 (Sy Stat Software Inc.; California, USA). One-way analysis of variance was used to evaluate COP_p_, COP_i_, cytokines, MAP and fluid balance between different treatment time points. If there was a significant difference between the mean values of the groups, an all pairwise multiple comparison procedure (Holm-Sidak or Dunn’s methods) was used to isolate the specific groups. Nonparametric tests for correlation calculations of continuous variables were performed by Spearman’s rank correlation test, and categorical variables were analyzed by the Mann–Whitney U-test. Continuous variables are expressed as mean ± SD, and categorical variables are expressed as counts and percentages. A *P*-value < 0.05 was considered statistically significant.

## Results

### Demographics

Twenty-nine neonates had moderate to severe HIE and were treated with hypothermia in the NICU during the study period. Seventeen of these patients (58%), 8 (47%) female, were included in the study. Twelve neonates could not be included due to lack of study personnel available for implanting wicks. A total of 13 out of 17 participating infants survived after 3 days of TH, and one infant developed necrotizing enterocolitis (NEC). One non-survivor had a congenital diaphragmatic hernia. The included patients did not have known septicemia, genetic or neuromuscular abnormalities and were not influenced by maternal chorioamnionitis. No patients were clinically characterized as edematous throughout TH. Gestational age (GA) varied from 35^+ 4^ to 42^+ 3^ weeks (mean GA of 39^+ 6^ weeks eq. 279 days), and average weight was 3464 g (range 2483–4710 g). One patient received TH despite having a GA below 36 weeks. There were no sex-related responses in included patients, and additional clinical characteristics are presented in Table 2.

**Table 2:** Study group characteristics of enrolled patients

Characteristics	*N* = 17
Gender: male, *n* (%)	9 (53)
Apgar score at 1 min, mean (range)	1 (0–5)
Apgar score at 5 min, mean (range)	2 (0–6)
Apgar score at 10 min, mean (range)	2 (0–6)
PROM, n (%)	3 (18)
Delivery mode, *n* (%)	
Emergency cesarean	9 (53)
Vaginal	8 (47)
SGA, n (%)	4 (26)
Seizures, requires anticonvulsant drug, *n* (%)	7 (41)
Referred from other hospitals, *n* (%)	6 (35)

*PROM* Premature rupture of membranes > 24 h, *SGA* Small for gestational age, indicates birth weight below 10th percentile for gestational age

### Fluid calculations, hemodynamic and laboratory variables during TH

Seven patients received inotropic support with dopamine as the primary vasopressor. MAP demonstrated a significant drop (*P* < 0.05) from 47.3 ± 6.9 mmHg at 7 h to 42.9 ± 8.2 mmHg at 25 h of life, but increased to 50.7 ± 5.3 when rewarming was initiated. A positive fluid balance of 4.1 ± 2.4 ml/kg/h 7 h after birth decreased significantly (*P* < 0.001) to 1.6 ± 1.9 ml/kg/h 13 h after birth and declined further to 1.0 ± 1.0 ml/kg/h until TH was completed. Urine output increased fourfold (*P* < 0.05) and nearly sevenfold (*P* < 0.05) from 7 to 25 and 73 h after birth (0.19 ml/kg/h to 0.85 ml/kg/h and 1.28 ml/kg/h respectively). Following TH, hemoglobin levels also decreased together with a transient decline in serum sodium levels between 7 and 25 h, whereas potassium and creatinine levels remained stable throughout the period. Serum glucose levels were reduced to approximately 50% during the first 24 h after birth. Albumin remained within the reference range [20], although levels significantly declined from 29 g/l to 26 g/l (*P* < 0.05) from the first 24 h to the 25–97-h period. Laboratory variables during TH and the first 24 h of rewarming are presented in Table 3.

**Table 3:** Biological data during therapeutic hypothermia and rewarming

Biological parameters	Therapeutic hypothermia, hours after birth					Rewarming
	7 h	13 h	25 h	49 h	73 h	97 h
pH	7.1 ± 0.2	7.3 ± 0.1	7.3 ± 0.1	7.3 ± 0.1	7.3 ± 0.1	7.3 ± 0.1
pCO_2_ (kPa)	6.5 ± 2.7	5.8 ± 1.7	5.5 ± 1.0	5.7 ± 1.0	6.0 ± 1.1	6.1 ± 1.2
BE (mmol/l) (−)	12.4 ± 5.1	6.5 ± 3.7	6.7 ± 3.2	5.8 ± 4.1	4.6 ± 2.9	3.5 ± 1.7
Hemoglobin (g/dl)	15.6 ± 2.2	15.4 ± 1.3	15.8 ± 1.8	15.1 ± 1.8	14.4 ± 1.6	13.5 ± 1.7
WBC (× 10^9^/l)	23.7 ± 13.2^1, 2^	24.8 ± 12.7^3, 4^	19.0 ± 7.9	14.9 ± 6.1	11.6 ± 5.5	10.7 ± 3.5
ANC (× 10^9^/l)	12.8 ± 10.4	15.5 ± 7.2^3, 4^	13.8 ± 6.3^5, 6^	10.1 ± 4.8	6.4 ± 2.2	6.9 ± 2.8
Lymph. (× 10^9^/l)	8.7 ± 5.5^1^	4.1 ± 2.7	3.3 ± 1.7	3.4 ± 1.5	2.9 ± 0.7	2.6 ± 0.7
Mono. (× 10^9^/l)	1.5 ± 1.2	2.1 ± 1.2^3, 4^	1.6 ± 0.9^6^	1.0 ± 0.6	0.7 ± 0.4	0.7 ± 0.4
Thrombocytes (× 10^9^/l)	217.0 ± 78.9	178.1 ± 57.8	158.9 ± 63.7	165.2 ± 80.3	143.3 ± 75.6	148.2 ± 75.5
CRP (mg/l)	15.6 ± 13.5	11.0 ± 13.5	10.7 ± 10.7	15.0 ± 12.4	23.6 ± 21.6	23.0 ± 28.7
s-glucose (mmol/l)	11.0 ± 4.8	10.1 ± 6.1	8.1 ± 6.6	5.7 ± 1.0	5.8 ± 2.3	5.3 ± 0.9
s-creatine (mcmol/l)	78.1 ± 16.1	86.9 ± 21.2	81.7 ± 32.1	86.5 ± 46.3	91.3 ± 67.9	85.0 ± 80.7
s-sodium (mmol/l)	135.8 ± 3.2	126.4 ± 32.2	133.9 ± 3.0	134.7 ± 4.0	135.2 ± 5.1	135.9 ± 6.2
s-potassium (mmol/l)	4.3 ± 0.7	4.2 ± 0.7	4.1 ± 0.8	4.0 ± 0.5	4.0 ± 0.6	4.2 ± 0.7
s-albumin (g/l)	28.8 ± 3.6^7^			25.7 ± 3.8		

All values represent the mean ± SD. *WBC* White blood cell count, *ANC* Absolute neutrophil count, *Lymph* Lymphocytes, *Mono* Monocytes, *CRP* C-reactive protein, ^1^*P* < 0.05 at 0–7 h vs. at 73–97 h, ^2^*P* < 0.05 at 0–7 h vs. at 49–73 h, ^3^*P* < 0.05 at 7–13 h vs. at 74–97 h, ^4^*P* < 0.05 at 7–13 h vs. at 49–73 h, ^5^*P* < 0.05 at 13–25 h vs. at 74–97 h, ^6^*P* < 0.05 at 13–25 h vs. at 49–73 h, ^7^*P* = 0.049 at 0–25 h vs. at 49–97 h

### Effect of TH on inflammatory markers

C-reactive protein showed a marginal increase from 16 mg/l to 24 mg/l between 49 and 73 h after birth (Table 3). Circulating total white blood cells (WBC) decreased significantly within the time points (7, 13, 25, 49, 73, and 97 h), as well as leukocyte subclasses, such as neutrophils, lymphocytes, and monocytes. There was a minor decline in the number of thrombocytes throughout TH (defined by platelet count < 150 ×  10^9^/l).

### Effect of TH on COP

An initial power-sample size evaluation based on *n* = (2 * (SD/delta))^2*k^ calculated *n* = 13 if a change in COP_p_ of 2.5 mmHg with 90% test strength and 5% level of significance was to be achieved. Similarly, a COP_p_ change of 1 mmHg or 2 mmHg need *n* = 40 and 20 respectively.

Therefore, n between 12 and 20 should be adequate to detect a 2–2.5 mmHg change of COP_p_.

**Fig. 1:**
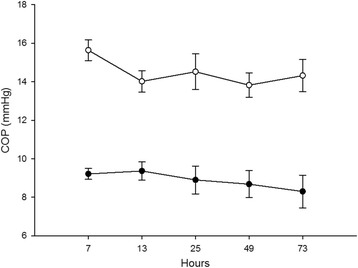
Colloid osmotic pressure and therapeutic hypothermia. Measured colloid osmotic pressure in serum (○) and interstitial fluid (●) plotted against duration of therapeutic hypothermia

A total of 22/68 wicks (32%) were discarded owing to blood contamination. Mean wick implantation time was 61 min. Mean COP_p_ and COP_i_ during TH were 14.5 ± 2.6 mmHg and 8.9 ± 1.9 mmHg, respectively. For COP_p_, we observed a decrease from 15.6 ± 1.9 mmHg at 7 h after birth to 13.8 ± 2.4 mmHg at 49 h of life, followed by a modest increase to 14.5 ± 2.6 mmHg at 73 h. For COP_i_, there was a small decline throughout TH from 9.2 ± 0.8 mmHg at 7 h to 7.8 ± 1.7 mmHg at 73 h (Fig. 1). However, none of the changes in COP_p_ or COP_i_ during TH were significant (*P* = 0.12 and 0.954, respectively). The pressure gradient, ΔCOP, between plasma and interstitium remained unchanged, although there was a tendency to increase during TH.

### Effect of TH on IF and serum levels of growth factors, cytokines, and chemokines

**Fig. 2:**
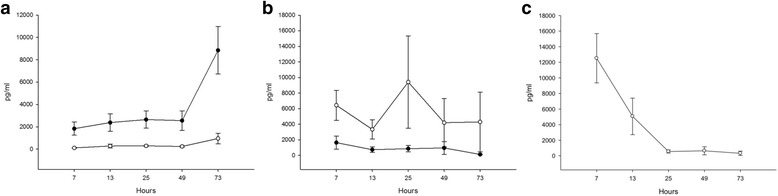
Cytokines and neonatal asphyxia. Median cytokines levels over time in serum (○) and interstitial fluid (●) during therapeutic hypothermia. X-axis refers to hours after initiation of therapeutic hypothermia, y-axis refers to concentration, pg/ml. **a**, Interleukin (IL)-1α **b**, IL-6. **c**, IL-10 (IF IL-10 not detectable)

In contrast to the minimal changes in COP, there were several alterations in levels of inflammatory mediators during TH. Compared with the measured values at 7 h, the serum IL-1RA levels significantly decreased between 49 and 73 h (*P* < 0.05). IL-6 levels were significantly decreased at 73 compared with levels at 7 h (*P* < 0.05), and IL-10 levels were significantly decreased at 25, 49, and 73 h compared with levels at 7 h (*P* < 0.05). The changes in these IF inflammatory parameters were very different from what we observed in the serum levels. IL-1α was the only IF cytokine with a significant (*P* < 0.05) increase throughout TH, with a rapid rise after 49 h. Conversely, IL-1RA levels were significantly decreased after 7 and 25 h of TH (*P* < 0.05). Additionally, higher serum IL-6 and IL-8 levels, compared with IF, were observed throughout TH. Changes in the remaining cytokine levels were small and non-significant. The mean levels of measurable cytokine concentrations in IF and serum at various time points during TH are presented in Table 4 and selected cytokines in Fig. 2.

**Table 4:** Measured cytokines in serum and interstitial fluid throughout therapeutic hypothermia

Cytokines		Therapeutic Hypothermia, hours after birth					
		7 h	13 h	25 h	49 h	73 h	Significance
IFN-γ (pg/ml)	Serum	26 ± 25	5 ± 65	NM	NM	NM	
	IF	24 ± 18	3 ± 25	39 ± 36	47 ± 59	28 ± 25	NS
IL-1α (pg/ml)	Serum	119 ± 41	287 ± 501	300 ± 280	246 ± 262	958 ± 945	NS
	IF	1840 ± 2034	2387 ± 2719	2652 ± 2682	2562 ± 3006	8845 ± 7055	*P* < 0.05^1^
IL-1RA (pg/ml)	Serum	38,845 ± 29,946	18,663 ± 17,076	8226 ± 9959	7917 ± 10,524	3069 ± 4132	*P* < 0.05^2^
	IF	1966 ± 1219	1141 ± 787	849 ± 1338	751 ± 421	2033 ± 2047	*P* < 0.05^3^
IL-6 (pg/ml)	Serum	6441 ± 6935	3343 ± 4747	9419 ± 4747	4204 ± 11,622	4297 ± 11,622	*P* < 0.05^4^
	IF	1646 ± 2663	742 ± 1098	868 ± 1325	964 ± 232	134 ± 104	NS
IL-8 (pg/ml)	Serum	3733 ± 2641	3025 ± 3590	2648 ± 4248	2059 ± 2567	1940 ± 2570	NS
	IF	678 ± 904	642 ± 904	1192 ± 2628	669 ± 1022	366 ± 534	NS
IL-10 (pg/ml)	Serum	12,519 ± 11,403	5059 ± 9073	557 ± 664	647 ± 1468	327 ± 665	*P* < 0.05^5^
	IF	NM	NM	NM	NM	NM	
IL-12p40 (pg/ml)	Serum	1018 ± 930	1064 ± 1088	1132 ± 1224	836 ± 875	NM	NS
	IF	NM	NM	NM	NM	NM	
MCP-1α (pg/ml)	Serum	51,752 ± 36,294	52,073 ± 43,265	63,297 ± 49,865	71,682 ± 42,825	54,762 ± 41,092	NS
	IF	3369 ± 2900	4367 ± 5604	6260 ± 8353	4381 ± 4062	5955 ± 3863	NS
TGFα (pg/ml)	Serum	64 ± 84	74 ± 98	53 ± 59	95 ± 114	73 ± 73	NS
	IF	NM	NM	NM	NM	NM	
TNFα (pg/ml)	Serum	503 ± 267	444 ± 246	449 ± 233	418 ± 213	505 ± 199	NS
	IF	19 ± 11	24 ± 19	24 ± 16	24.3 ± 18	37 ± 17	NS
VEGF (pg/ml)	Serum	4282 ± 3168	5103 ± 3309	1597 ± 1055	2514 ± 1545	3663 ± 1391	NS
	IF	1597 ± 1631	2654 ± 3044	717 ± 1071	1465 ± 2305	NM	NS

All values represent mean ± SD. *IF* Interstitial fluid, *NM* not measurable, *NS* no statistical significant difference. ^1^Significance between 73 h and all other time points; ^2^significance between 73 and 49 h and 7 h; ^3^significance between 25 and 7 h; ^4^significance between 73 and 7 h; ^5^significance between 73, 49, and 25 h and 7 h

### Correlations between changes in IF and serum cytokine levels, and clinical and imaging parameters

We found a significant correlation between decreased MAP and increased IL-1α in the IF between 7 and 49 h, and a non-correlating rapid rise in interstitial IL-1α and elevated MAP from 49 to 73 h. We also observed significant negative correlations between IF IL-1α levels at 7 h and later measurements of COP_p_ (12, 24, and 72 h) and COP_i_ (48 h). Additionally, the IF IL-1α levels at 13 and 25 h were associated with COP_p_ at 73 h. Although serum levels of IL-1RA and IL-10 significantly decreased during TH, there were no significant correlations with MAP, COP_p_, COP_i_, or fluid balance for these cytokines. For all other cytokines, there was no association with MAP, COP_p_, COP_i_, or fluid balance, as well as no correlations between cytokine levels and clinical variables.

## Discussion

In the present study, HIE neonates were treated with TH and experienced a reduced COP_p_ similar to previously reported in asphyxiated newborns [21]. The interstitial COP from TH treated neonates was nearly halved compared to previous reports from healthy children [14], and during TH a non-significant decrease was found in both COP_p_ and COP_i_ throughout the various treatment time points. In the IF, the pro-inflammatory IL-1α showed increased levels during hypothermic treatment, which coincided with decreased total WBC count and no clinical or laboratory signs of edema formation.

Neonates that experience scarce spontaneous ventilation, inadequate heart rates, and low Apgar scores at 1–5 and 10 min after birth are often resuscitated and in need of intensive care treatment. In addition to respiratory support, great care is taken to regulate circulatory homeostasis with adequate intravascular volume and efficient organ perfusion. Although a clinician may control the volume of administered fluid, the ability to dictate its pathways is challenging owing to deviation in blood pressure, circulating blood volume, interstitial accumulation of fluid and capillary permeability. Among the forces that alter passage of fluid from blood vessels to the interstitium, COP_p_ and COP_i_ are most easily examined in the plasma (equal to serum) and by extracting IF from subcutaneously implanted wicks. The endothelial glycocalyx, a multicomponent luminal network of membrane bound proteoglycans and glycoproteins covering the endothelial cells, creates a COP gradient close to the barrier of filtration. COP_i_, a marker of glycocalyx COP (COP_g_), works close to the endothelium border and is thought to mirror COP_g_ under normal filtration rates [22]. COP_p_ in healthy adults (25 mmHg) [17, 23] is reported equal to children [14] but higher than in term [24]- and pre-term neonates [15] (20 mmHg and 15 mmHg, respectively). Sick neonates, regardless of maturity, have an even more decreased COP_p_ [15, 24, 25]. Sussmane et al. showed that COP_p_ in healthy infants from 1 to 11 months is in proportion to adult values, with an anticipated COP_p_ increase during the first month of life [26]. Throughout TH treatment in the present study, the mean COP_p_ (14.3 ± 2.6 mmHg) coincides with earlier observations of lowered plasma COP in sick newborns [15]. Although there were no significant changes in COP in serum during TH, the decline in COP_p_ during the first 13 h was most likely due to simple dilution of plasma proteins. A possible explanation is the initial treatment with intravenous fluid resuscitation and subsequent stabilization in the NICU. The following fluid restriction will result in a more constant COP_p_ (Fig. 1). These results correspond with the significantly increased positive fluid balance during the first 7 h after birth compared with the remaining TH period (*P* < 0.01) and a transient hyponatremia of 126 mmol/l serum sodium at 13 h after birth. Skin vasoconstriction due to whole body cooling, as well as reduced urine output, elevated serum creatinine, and mechanical ventilation, are potential reasons for excess fluid. Such fluid accumulation occurred despite our adherence to the recommended reduced daily fluid intake. More novel is the finding of a reduced COP_i_ of 8.9 ± 1.9 mmHg compared with COP_i_ from children between 2 and 10 years (13.9 ± 3.5 mmHg) [14], and a corresponding ΔCOP (i.e., COP_p_ – COP_i_) nearly constant during TH. A relatively balanced COP gradient together with a reduced serum albumin, suggests that other plasma proteins compensate in maintaining COP_p_ since the patients were only, to a small extent, administered crystalloids and blood products. Unfortunately, there was not enough material to measure total protein and albumin concentration in the IF owing to protocol restrictions. The significant decline (*P* < 0.05) in serum albumin from 29 to 26 g/l with stable COP_p_ between the first day and the last 2 days of TH may indicate capillary hyperpermeability propelled by the asphyxia event inducing an inflammatory cascade [11]. Other macromolecules that replace plasma albumin or reduced circulatory fluid would maintain an unaltered COP_p_. Conversely, reduced liver function with less synthesized albumin and restricted fluid access would produce a similar situation.

The present results showing albumin within the reference range, although reduced during TH, may be due to a reduced inflammatory stimulus as a result of hypothermia [27] or increased lymphatic fluid removal, although the latter was shown to be less likely in sedated and immobilized dogs [28].

According to the Starling equation, increased capillary filtration coefficient (CFC) (proportional to hydraulic conductivity (L_p_) and the capillary area accessible for fluid shift) and/or elevated capillary hydrostatic pressure (Pc), as well as a decline in the reflection coefficient (σ), may increase fluid extravasation. Capillary pressure is dependent on arterial pressure and venous pressure, together with pre- and post-capillary resistance [29]. Michel et al. demonstrated increased permeability due to elevated L_p_ after mild thermal injury, which expressed a biphasic course and diminished after several hours [30], whereas Lundblad et al. showed that endotoxemia in cat skeletal muscle resulted in reduced σ and significantly increased CFC [31]. Whether asphyxia and inflammation stimulates changes triggering barrier disruption owing to altered L_p_ or σ is uncertain, as well as how TH affects these parameters.

MAP, which decreased during the first 25 h of TH, is dependent on peripheral resistance and cardiac output, the latter known to be reduced during asphyxia and TH [32]. Consequently, an expected increased peripheral resistance, primarily post-capillary, takes place after TH initiation and results in increased Pc (given unaltered CFC and σ), thereby forcing extravasation to the interstitium. A small, but detectable, fall in COP_i_ compared with COP_p_ is maintained by removal of albumin or other proteins comparable in size from the interstitium. The reduced COP_i_ and COP_p_ during TH in asphyxiated neonates and the ability of TH to oppose changes in capillary filtration by COP in IF is an edema preventive mechanism demonstrated in animal [33] and human [34] studies.

Two thirds of patients in the present study developed mild thrombocytopenia, which is associated with birth asphyxia [35] as well as TH activation and aggregation of platelets with potential thrombus formation [13]. The reduced platelet count as a result of hypothermia is thought to be protective from microvascular slugging and related to increased blood viscosity and vasoconstriction; the patients in the present study exhibited no vascular incidents, with exception of one patient who required platelet transfusion and surgery owing to NEC during TH.

Results from the present study showed decreased levels of IL-6, a pleotropic cytokine, in IF compared with serum, which could be due to negligible tissue damage and subsequent inflammation; this supports the validity of the wick technique as a less traumatic method for sampling of IF. A significant reduction in WBC count with neutropenia was also observed along with a delayed initiation of CRP response. This was in accordance with recent observations of non-sepsis-risk neonates treated with TH [36]. Leukopenia with reduced IL-10 is associated with better outcome in animal models of brain injury and in human adult stroke patients; the observed functional immune compromise may be due to bone marrow suppression and reduced leucocyte release [10]. Conversely, a prolonged leukopenia that does not recover after rewarming, as we observed, is associated with a poor prognosis at 12 months [37], although treatment outcome was beyond the scope of this trial to measure.

Endothelial dysfunction is associated with perinatal asphyxia [38], and the concomitant acute inflammation from ischemia-reperfusion injury triggers a compulsory formation of intracellular gaps owing to activation of the actin-myosin system with increased permeability. To the best of our knowledge, increased levels of IL-1α in IF from skin, which suggests local cytokine production, has never been measured in neonates. The finding of an amplified IL-1α in the IF throughout TH treatment, suggests an up-regulation from keratinocytes and other cells in the subcutis [39]. Whether IL-1RA, which decreased during the first 49 h of TH, antagonized IL-1α initially is uncertain. Although recombinant IL-1α injected intravenously into rabbits exerts a rise in temperature [39], it remains unclear whether the increased IL-1α in IF was due to asphyxia or potentiated by the hypothermic environment. The correlation between increased IL-1α and decreased MAP was not obvious, although TH-increased post-capillary resistance may be influenced by inflammation-affected endothelium and the resulting increased extravasation resulting in a lowered COP_i_. Mild hypothermia can be demonstrated by a lowered secretion of IL-10 in cell culture [40], as well as by interleukin expression in hypothermic neonates, although levels are initially higher during TH [37]. This was in line with the findings from the present study. The anti-inflammatory effects of TH on the vascular endothelium remain uncertain and not well studied, although a recent report showed better outcomes in neonates treated with shortened TH for 49 h, as well as down-regulation of serum IL-6, IL-8, and IL-10 [37].

The present study has some limitations. The most important of these limitations is that an unusually high number of wicks had to be discarded due to blood contamination, thus limiting the aggregate of measurements with each time-period during TH and thereby the power of the analysis at some data points. Unusable wicks represented nearly 50% of extracted wicks at 7 and 73 h after birth. Time from birth to initiation of TH was not identical within the group and may have influenced the relationship between biological parameters and time periods of TH, although less likely since body temperature were kept between 34 and 36 °C after initial stabilization. Also, the results presented are limited to the experience of one tertiary NICU and may not be generalizable. The osmolality of fluids intravenously administered during TH was not calculated, although it is less likely that crystalloids have a major impact on intravascular tonicity. Daily weight was not measured owing to the attached technical equipment. Therefore, potential fluid loss from respiratory evaporation or obvious fluid accumulation during TH was undetected. The clinical evaluation of edematous skin, together with fluid calculations and measurements of vascular tonicity, was performed by the attending physician and variations in fluid treatment may have occurred. Furthermore, the degree of asphyxia is difficult to interpret with altered physiology in response to both hypoxic ischemia and TH.

## Conclusions

Neonates treated with TH have a decreased, but stable, COP_p_ and a compensatory low COP_i_. This results in reduced vascular filtration with a COP gradient counteracting fluid flux from the capillaries to the interstitium, which likely functions as an edema-preventing mechanism. The decrease in MAP and reduced positive fluid balance during the first 24 h of TH, as well as no observed clinical edema, supports this assumption. Furthermore, hypothermia induces an expected low WBC and platelet count in neonates without any risk factors for infection. This is likely part of an inherent asphyxia protection system together with a fairly balanced local and systemic cytokine production. The results presented in this study provide novel and valuable information concerning tissue cytokine production during TH.

## Abbreviations

CFC: Capillary filtration coefficient
COP: Colloid osmotic pressure
COP_i_: Interstitial colloid osmotic pressure
COP_p_: Plasma colloid osmotic pressure
GA: Gestational age
HIE: Hypoxic ischemic encephalopathy
IF: Interstitial fluid
IL: Interleukin
L_p_: Hydraulic conductivity
MAP: Mean arterial pressure
MRI: Magnetic resonance imaging
NEC: Necrotizing enterocolitis
NICU: Neonatal Intensive Care Unit
Pc: Capillary hydrostatic pressure
TH: Therapeutic hypothermia
WBC: White blood cells
